# Incident Duration Modeling Using Flexible Parametric Hazard-Based Models

**DOI:** 10.1155/2014/723427

**Published:** 2014-11-04

**Authors:** Ruimin Li, Pan Shang

**Affiliations:** Institute of Transportation Engineering, Department of Civil Engineering, Tsinghua University, Heshanheng Building, Tsinghua, Beijing 100084, China

## Abstract

Assessing and prioritizing the duration time and effects of traffic incidents on major roads present significant challenges for road network managers. This study examines the effect of numerous factors associated with various types of incidents on their duration and proposes an incident duration prediction model. Several parametric accelerated failure time hazard-based models were examined, including Weibull, log-logistic, log-normal, and generalized gamma, as well as all models with gamma heterogeneity and flexible parametric hazard-based models with freedom ranging from one to ten, by analyzing a traffic incident dataset obtained from the Incident Reporting and Dispatching System in Beijing in 2008. Results show that different factors significantly affect different incident time phases, whose best distributions were diverse. Given the best hazard-based models of each incident time phase, the prediction result can be reasonable for most incidents. The results of this study can aid traffic incident management agencies not only in implementing strategies that would reduce incident duration, and thus reduce congestion, secondary incidents, and the associated human and economic losses, but also in effectively predicting incident duration time.

## 1. Introduction

Traffic incidents are the primary causes of nonrecurrent traffic congestion on intercity expressways and arterial networks in cities [1, 2]. Many Advanced Traffic Incident Management (ATIM) systems have been deployed all over the world in the past two decades to reduce traffic incident duration and congestion level. The reliable estimation as well as prediction of traffic incident duration in real-time is necessary, albeit challenging, for the efficient operation of ATIM systems.

Incident duration, which can be defined as the time difference between incident occurrence and incident site clearance [3–5], includes four time intervals or phases [6]: (1) incident detection/reporting time, (2) incident preparation/dispatching time, (3) travel time, and (4) clearance/treatment time.

This study investigates the influences of various traffic incident characteristics, such as temporal, road, incident-related, and environmental characteristics, on incident duration time using parametric hazard-based models and flexible parametric hazard-based duration models, to provide more suitable distribution for the base hazard function. The dataset used in this study was extracted from the Incident Reporting and Dispatching System in Beijing, and it contains the characteristics and duration times of incidents that occurred on the 3rd Ring expressway mainline in 2008.

This paper begins with a literature review about previous research on incident duration analysis and prediction. This review is followed by details on flexible parametric hazard-based model development. Next, the used data is described with the use of descriptive analyses of incident duration time and incident characteristics. The model estimation results and model parameter interpretation are then presented. This paper concludes with a summary of findings and directions for future research.

## 2. Literature Review

Over the past few decades, many studies have been conducted to investigate appropriate approaches and techniques for the estimation and prediction of traffic incident duration time, mainly on freeways. The most typical approaches include (1) regression methods [3, 7–9], (2) Bayesian classifier [10–12], (3) Decision trees and Classification trees [13, 14], (4) neural networks [15–17], (5) the discrete choice model [18], (6) the structure equation model [19], (7) probabilistic distribution analyses [20, 21], (8) support/relevance vector machines [22], and (9) hybrid methods [23]. These studies on traffic incident duration modeling have been summarized elsewhere [24, 25].

Several kinds of hazard-based models have been recently used to estimate the factors affecting traffic incident duration/clearance time or predict traffic incident duration/clearance time. The majority of studies on incident duration analysis have used parametric hazard-based models, that is, accelerated failure time (AFT) models, because of the following reasons: (1) the baseline hazard rate contributes to the understanding of the natural history of the incident through the manner in which the hazard rate changes over time; and (2) the AFT model allows for the estimation of an acceleration factor that can capture the direct effect of a specific factor on survival time [26].

In a parametric model application, one must know the correct duration time distribution, which can be selected by using measures such as likelihood [4, 27] or Akaike's Information Criterion (AIC) to assess the goodness of fit [28, 29]. In the context of traffic incident duration, specific hazard distributions are suggested by empirical and theoretical analyses using different incident datasets with different incident types and locales. Previous studies have noted various distributions of incident duration, such as log-normal distribution, log-logistic distribution, Weibull distribution, and generalized F distribution.

Studies have revealed that the distribution of incident durations can be viewed as log-normal [20, 21]. A different study [5] that focused on the South Korean freeway system indicated that log-normal is an acceptable, but not the best, distribution for traffic durations.

Other researchers have found that the log-logistic distribution is best for traffic incident duration/clearance time. Jones et al. [30] used AFT models with log-logistic distribution on freeway incident records in Seattle to investigate the factors affecting traffic incident duration time. Chung [31] used the log-logistic AFT model to develop a traffic incident duration time prediction model; the resulting mean absolute percentage error (MAPE) showed that the developed model can provide a reasonable prediction based on a two-year incident duration dataset drawn from the Korea Highway Corporation on 24 major freeways in Korea. Using another dataset obtained from the Korea Highway Corporation, the log-logistic AFT model has also been used to analyze the critical factors affecting incident duration [5]. Qi and Teng [32] developed an online incident duration prediction model based on a log-logistic AFT model. Hu et al. [33] used a log-logistic AFT model to predict incident duration time for in-vehicle navigation systems based on Transport Protocol Experts Group data in London and obtained a reasonable result. Wang et al. [29] estimated traffic duration times by using a log-logistic AFT model based on traffic incidents occurring on a freeway in China.

The Weibull distribution has also been used in previous studies. Nam and Mannering [4] studied three duration phases (i.e., detection/reporting, response, and clearance times), and the results revealed that the Weibull AFT model with gamma heterogeneity is appropriate for detection/reporting and response time, whereas the log-logistic AFT model is appropriate for clearance time. Kang and Fang [34] used the Weibull AFT model to predict traffic incident duration time in China. To test the goodness of fit, Alkaabi et al. [35] used the Weibull AFT model without gamma heterogeneity to analyze traffic incident clearance time in Abu Dhabi, United Arab Emirates. Tavassoli Hojati et al. [28] studied three kinds of traffic incidents, and the results showed that Weibull AFT models with random parameters were most suitable for modeling the durations of crashes and hazards incidents, and a Weibull model with gamma heterogeneity was appropriate for modeling the duration of stationary vehicle incidents.

To find a more appropriate distribution for traffic incident duration, Ghosh et al. [27] used generalized F distribution, which includes a number of the most commonly used distributions in parametric hazard-based models, to assess the effects of certain factors on incident clearance times. The results showed that generalized F distribution provided the best fit for the incident clearance time data used in that study.

The chi-square results of another study showed that both the Weibull and log-normal stochastic models do not adequately describe the clearance time values for all incidents [9]. The histogram of incident clearance times with various characteristics showed different shapes.

Commonly used distributions impose restrictions on the shape of the hazard function, and the distribution of traffic incident duration time is diverse. For example, the distribution of traffic incident duration times may be neither Weibull nor log-logistic; that is, simpler parametric models may not be flexible enough to adequately represent the hazard function and capture the underlying shape of the data. Therefore, more flexible models [36] are needed to greatly extend the range of hazard distributions that can be estimated [37]. In the past decade, various more flexible distributions [36, 38, 39] have been used in hazard-based models.

The factors that significantly affect incident duration time vary with the dataset and the various available variables. The various factors identified in previous studies generally included the following: temporal factors (e.g., time of day, day of week, and peak hour versus nonpeak hour), incident characteristics (e.g., different collision types, involving trucks, buses, taxis, or none of these), environmental conditions (e.g., rainfall, fog), roadway geometry, traffic flow conditions (e.g., congestion versus noncongestion), and operational factors.

Using a dataset consisting of 2851 traffic incident records obtained from the 3rd Ring expressway mainline in Beijing, this study assesses the effects of various distributions on a hazard-based model used to analyze incident duration time on the basis of the selected measure of fit. After the performances of various models are compared, the best model is used to investigate the relationship between various factors and traffic incident duration time as well as to predict traffic incident duration time.

## 3. Flexible Parametric Model

When a traffic incident occurs, travelers and traffic operators are concerned over the length of time between the reporting and clearance of the incident, as well as the probability that the incident will end in the next time period *t* + Δ*t*, given that it has lasted for a specific time *t*. Probabilities that change over time are ideally suited for hazard-based analysis [40].

First, the cumulative distribution function of a hazard-based model is defined as follows:

(1) $$\begin{array}{c} F\left(t\right)=\mathrm{Pr}\left(T<t\right), \end{array}$$

where *Pr*⁡() denotes probability, *T* is a continuous random variable, and *t* is a specified time. This function specifies the probability that an incident will end before transpired time *t*. *F*(*t*) is also known as the failure function.

Another basic function in hazard-based modeling is the survivor function *S*(*t*), which is expressed as follows:

(2) $$\begin{array}{c} S\left(t\right)=\mathrm{Pr}\left(T\geq t\right)=1-\mathrm{Pr}\left(T<t\right)=1-F\left(t\right). \end{array}$$

This function provides the probability that an incident is equal to or longer than the specified time *t*.

The cumulative hazard function *H*(*t*) can be related with the survival function by using the well-known mathematical formula *H*(*t*) = −ln⁡*S*(*t*). Based on the log cumulative hazard scale, with a covariates vector *z*, the proportional hazards model can be expressed as follows:

(3) $$\begin{array}{c} \ln\left\{H\left(t\;|\;z_{i}\right)\right\}=\ln\left\{H_{0}\left(t\right)\right\}+\beta^{T}z_{i}. \end{array}$$

Given *H*(*t*) = −ln⁡*S*(*t*), (3) can be rewritten in the following equivalent form [37]:

(4) $$\begin{array}{c} \ln\left\{-\ln S\left(t\;|\;z_{i}\right)\right\}=\ln\left\{-\ln S_{0}\left(t\right)\right\}+\beta^{T}z_{i}, \end{array}$$

where *S*_0_(*t*) = *S*(*t*∣0) is the baseline survival function and *β* ^*T*^ is a vector of parameters to be estimated for covariates *z*.

Equation (4) can be generalized to [36]

(5) $$\begin{array}{c} g_{\theta }\left\{S\left(t\;|\;z_{i}\right)\right\}=s\left(x,\gamma \right)+\beta^{T}z_{i}, \end{array}$$

where *g*_*θ*_(·) is a monotonic increasing function depending on a parameter *θ*, *x* = ln⁡*t* and *γ* is an adjustable parameter vector.

Royston and Parmar [36] took *g*_*θ*_(·) to be Aranda-Ordaz's function:

(6) $$\begin{array}{c} g_{\theta }\left(s\right)=\ln\left(\frac{s^{-\theta }-1}{\theta }\right), \end{array}$$

where *θ* > 0.

The limit of *g*_*θ*_(*s*) as *θ* tends toward 0 is ln⁡(−ln⁡*s*), so that when *θ* = 0, the proportional hazards model can be expressed as *g*_*θ*_{*S*(*t*∣*z*)} = ln⁡(−ln⁡(*S*(*t*∣*z*))). When *θ* = 1, the proportional odds model can be expressed as *g*_*θ*_{*S*(*t*∣*z*)} = ln⁡(*S*(*t*∣*z*)^−1^ − 1). When *g*_*θ*_(·) is defined as an inverse normal cumulative distribution function, the probity model can be expressed as *g*_*θ*_{*S*(*t*∣*z*)} = −Φ^−1^(*S*(*t*∣*z*)), where Φ^−1^() is the inverse normal distribution function.

As flexible mathematical functions, splines are defined by piecewise polynomials, but with some constraints to ensure that the overall curve is smooth; the split points at which the polynomials join are known as knots [41]. Cubic splines are the most commonly used splines in practice. Restricted cubic splines [42] are used in this study with the restriction that the fitted function is forced to be linear before the first knot and after the final knot. Restricted cubic splines offer greater flexibility than standard parametric models in terms of the shape of the hazard function [37]. Restricted cubic splines with *m* distinct internal knots, *k*_1_,…, *k*_*m*_, and two boundary knots, *k*_min⁡_ and *k*_max⁡_, can be fit by creating *m* + 1 derived variables. A restricted cubic spline function is defined as follows:

(7) $$\begin{array}{c} s\left(x,\gamma \right)=\gamma_{0}+\gamma_{1}x+\gamma_{2}v_{1}\left(x\right)+\cdots +\gamma_{m+1}v_{m}\left(x\right). \end{array}$$

The derived variables *v*_*j*_(*x*) (also known as the basis function) can be calculated as follows:

(8) $$\begin{array}{c} v_{j}\left(x\right)={\left(x-k_{j}\right)}_{+}^{3}-\lambda_{j}{\left(x-k_{\min}\right)}_{+}^{3}-\left(1-\lambda_{j}\right){\left(x-k_{\max}\right)}_{+}^{3}, \end{array}$$

where for *j* = 1,…, *m* *λ*_*j*_ = (*k*_max⁡_ − *k*_*j*_)/(*k*_max⁡_ − *k*_min⁡_) and (*x* − *a*)_+_ = max⁡(0, *x* − *a*).

The baseline distribution is Weibull or log-logistic with *m* = 0, meaning that no internal and no boundary knots are specified; that is, *s*(*x*, *γ*) = *γ*_0_ + *γ*_1_ *x* [36].

Parameters *γ*, *β* can be estimated by using the maximum likelihood [36], and confidence intervals can be estimated from the Hessian at the maximum.

## 4. Numerical Application

### 4.1. Data Description

The studied incident dataset was obtained from the Incident Reporting and Dispatching System (IRDS) for the Beijing metropolitan area, which covers all kinds of roads. The IRDS database in the traffic control center contains all types of incidents that were reported to the control center, regardless of whether the common incident response units (i.e., traffic police) had responded to these incidents. According to previous studies [4, 27, 35], the roads where incidents occur have significant influences on traffic incident duration, presumably because of various road characteristics and other unobserved factors. However, at present, we are unable to acquire detailed information on all of the roads in Beijing. Therefore, in this study, only the incident data for the 3rd Ring Road mainline are chosen to aid in reducing the influence of different roads on traffic incident duration time.

From the IRDS database, the time of different incident duration phases can be calculated, including preparation time, travel time, clearance time, and total time, which is the sum of the first three phases. The final studied incident dataset contains 2851 incident records for a one-year period (2008), with each incident duration phase being equal to or greater than one minute.  Table 1 provides the summary statistics information for the incident dataset used in this study.

The positive skewness value, as well as the minimum, maximum, and mean values, indicates that the tail on the right of all four of these distributions is longer than that on the left side; that is, the distributions are right long tailored. The higher kurtoses of the different duration phase data mean that much of the variance is the result of infrequent extreme deviations, suggesting that infrequent extreme values are present in the dataset. Taking travel time as an example, the longest travel time is 245 min, but the second longest is only 114 min. Such outliers can present difficulties both in developing estimated models and in predicting duration time.

Some candidate variables related to temporal characteristics, incident and traffic condition, and so on, can be extracted from the IRDS. This study analyzes the variables affecting traffic incident duration time to develop incident duration time prediction models, which would be helpful in incident management. Therefore, this study considered and used only specific candidate variables (shown in Table 2) that can be obtained immediately after an incident has been reported to the traffic control center.

As mentioned above, traffic incident duration includes four time intervals [6]. However, traffic incident duration may be divided mechanically, and the former interval may affect the next interval. Thus, we take preparation time and travel time as effective factors to be examined for later intervals. Table 2 shows the candidate variables used in this study.

### 4.2. Distribution Choice and Model Development

To choose a spline function, the number and position of the knots, that is, the number of degrees of freedom (d.f.), must be decided. The optimal (optimized) knot position does not appear to be critical for a good fit and may even be undesirable, in that the fitted curve may follow the small-scale features of the data too closely [37]. A previous study [36] suggested that knot positions are based on the empirical centiles of the distribution of log time. In terms of the number of knots, one study suggested [37] that a two- or three-d.f. spline model would be a reasonable initial or default choice for smaller datasets, whereas five or six d.f. would be necessary with larger datasets.

As mentioned above, previous studies have found that several distributions can be used for the hazard-based model to analyze or predict traffic incident duration time. Thus, in the present study, except for the flexible parametric model based on restricted cubic splines, four other commonly used distributions are also used as candidates in parametric hazard-based models, namely, Weibull, log-normal, log-logistic, and generalized gamma.

Informally, the AIC, Bayesian Information Criterions (BIC), or others [35] can be used as criteria for choosing the “best-fit” model. This study used BIC, which is expressed as follows:

(9) $$\begin{array}{c} \text{BIC}=-2l+\log\left(n\right)d, \end{array}$$

where *l* is the maximized value of the log-likelihood for a given model, *n* is the number of the observations, and *d* is the number of free parameters to be estimated.

### 4.3. Selected Model

In this study, 17 candidate different models with different distributions were used to fit the data. The best-fit model was chosen according to the BIC value. For each incident phase, these 17 models include AFT model with Weibull, log-logistic, generalized gamma with or without frailty, and flexible parametric model with 1 to 10 degrees of freedom.  Table 3 lists the BIC value of each model. The best-fit model is used to analyze the effective factors of each incident and predict the time of each incident phase.

As shown in Table 3, the AFT hazard-based model with generalized gamma distribution is the best-fit model for preparation time and total time, the flexible parameter model with six knots (five degrees of freedom) is the best-fit model for travel time, and the log-logistic model is the best-fit model for clearance time.

### 4.4. Effective Factor Analysis

The best-fit model can be used to analyze the effect of effective factors for each incident phase.  Table 4 shows the regression coefficients of different factors and the percentage change for each incident phase.

#### 4.4.1. Preparation Time

Preparation time is the difference between the time when operators received the incident report call and the time when the incident response team members were dispatched.

*Temporal Characteristics*. Incidents that occurred in summer and autumn were associated with longer preparation time. When the preparation time in spring was considered as the reference, the preparation time in summer and autumn had 13.31% and 16.88% extra time more than that in spring, respectively. The reason might be due to that fact that more incidents occurred in the roads in summer and autumn; thus, the average incident response of available response teams for each incident was less, which might have resulted in a longer preparation time. 

*Incident Characteristics*. The incidents that included overturned vehicles had shorter preparation time than more common crashes. Given that incidents involving overturned vehicles may include fatality or injuries, these incidents were therefore treated as the most important cases to respond to and required the response team to prepare as soon as possible. The incidents involving taxis likewise needed a longer preparation time and used 4.6% of extra time for preparation. 

*Geographic Characteristics.* Incidents that occurred far from the city center were associated with shorter preparation time. As the distance of the incident site from the city center increased by 1 km, the preparation time became 4.23% shorter. This phenomenon may be because more incidents occur near the city center as a result of increased traffic flow, and dispatching the incident response team near the city center can be difficult. By contrast, fewer incidents occur in the suburbs, allowing the operators to easily dispatch the response team and resulting in less preparation time. 

Road congestion can be a significant factor in preparation time. The preparation time was 5.82% shorter when the road was congested than when it was uncongested. When an incident occurred in a congested road, the harmful effect was great; thus, the problem needed to be solved quickly and the operators had to prioritize this incident.

#### 4.4.2. Travel Time

Travel time is the difference between the time when the incident response team members received the dispatch order and the time they arrived at the incident site. 

*Temporal Characteristics*. The travel time for incidents that occurred in the first shift of the day was less difficult to finish yet was longer because the incident response teams were fewer for this shift than for the second shift. The travel time for incident response teams to arrive at the incident site was therefore longer. Incidents that occurred in autumn were associated with longer travel time. 

*Incident Characteristics*. Incidents that involved bicycles or pedestrians, or incidents of collision with stationary objects, had longer travel time than common crashes. Incidents involving taxis or buses had shorter travel time. The latter type of incidents might be more severe and have more harmful effects. Delays in solving these incidents may lead to severe congestion. Thus, the incident response teams considered these incidents as the most important cases and would quickly travel to the incident sites. 

#### 4.4.3. Clearance Time

Clearance time is the difference between the time when the incident response team arrived at the incident site and the time when the incident site was cleared. 

*Temporal Characteristics.* Incidents that occurred in the first shift of the day were associated with longer clearance time, that is, approximately 73.84% longer than the second shift, because of two possible reasons. First, the incidents that occurred in first shift were usually more severe because vehicles ran faster during this time. Second, the lighting on the incident sites might not be sufficient during the night, resulting in a longer time to clean these sites. 

*Incident Characteristics.* The incidents involving overturned vehicles had longer clearance time than common crashes. These incidents required more than 163% of the clearance time because the overturned vehicles could not be driven, thereby requiring the assistance of a tow truck, which in turn increased the clearance time. This fact presents the challenge of how to clear overturned vehicles effectively. 

*Geographic Characteristics*. Incidents that occurred far from the city center were associated with longer clearance time; that is, as the distance of the incident site from the city center increased by 1 km, the clearance time became 29.04% longer. Moreover, when the road was congested, clearance time was 19% longer. Road congestion thus significantly affects the clearance time. 

*Preparation Time*. Preparation time affected the clearance time. In this study, when the preparation time of the incident was longer, the clearance time was shorter. Why longer preparation time results in shorter clearance time requires further investigations. 

#### 4.4.4. Total Time

Total time is the sum of the preparation time, travel time, and clearance time. 

*Temporal Characteristics*. Incidents that occurred during the first shift of the day were associated with longer total time. The reason may be that when the incidents occurred in the first shift (i.e., from 10 PM to 6 AM), most of the incidents were severe because of the poor lighting, higher speed, and other reasons, thereby requiring more clearance time. Thus, the incidents that occurred during the first shift required longer total time.

Incidents that occurred in winter were also associated with longer total time. In winter, Beijing may experience snow, and the temperature is low. Such poor weather conditions make all of the work more difficult, thus increasing the total time in winter. 

*Incident Characteristics*. Incidents involving bicycles or pedestrians, collisions with stationary objects, or overturned vehicles had longer total time than common crashes. These types of incidents were severe, and the incident response teams and police had more responsibilities. Thus, the total time was longer. 

*Geographic Characteristics*. Incidents that occurred far from the city center were associated with longer total time. The total time was 14.45% longer as the distance of the incident site from the city center increased by 1 km. Road congestion can significantly affect total time. The roads leading to such sites could be congested, suggesting that incidents that occurred on these roads required a longer total time. Under a congested condition, arriving at the incident site and clearing the area would therefore require longer time. 

For the results of 3rd ring mainline, different factors had different effects on incident duration. For example, distance from the city center significantly affects preparation time, clearance time, and total time but does not affect travel time. According to these results, fitting the best model for each incident duration phase separately when analyzing traffic incident duration is necessary.

## 5. Prediction

The dataset used in this study was divided into two groups. One group contained 2/3 of the data and was used to estimate the best-fit model. Another group contained 1/3 of the data and was used to test the prediction accuracy.

To investigate the accuracy of predictions, three indices, namely, root mean squared error (RMSE), mean absolute error (MAE), and mean absolute percent error (MAPE), were calculated to compare observed and predicted results. MAE is expressed as follows:

(10) $$\begin{array}{c} \text{MAE}=\frac{1}{n}\overset{n}{\underset{i=1}{\sum }}\left|A_{i}-P_{i}\right|. \end{array}$$

RMSE is expressed as follows:

(11) $$\begin{array}{c} \text{RMSE}=\sqrt{\frac{1}{n}\overset{n}{\underset{i=1}{\sum }}{\left(A_{i}-P_{i}\right)}^{2}}. \end{array}$$

MAPE is a summary measure widely used for evaluating the accuracy of prediction results and can be expressed as follows:

(12) $$\begin{array}{c} \text{MAPE}=\frac{1}{n}\overset{n}{\underset{i=1}{\sum }}\left|\frac{A_{i}-P_{i}}{A_{i}}\right|, \end{array}$$

where *A*_*i*_ denotes the actual value for the *i*th observation and *P*_*i*_ refers to the predicted value for the *i*th observation. Lower values of RMSE, MAE, and MAPE correspond to the higher accuracy of the prediction model. Tables 5 and 6 show the MAE, RMSE, and MAPE calculation results for models used for the prediction dataset.

As shown in Tables 5 and 6, the preparation time predicted by using the evaluation index proposed by Lewis [43] was reasonable; however, the other predictions were inaccurate. For different duration ranges, the RMSE and MAPE were relatively low for near average durations, that is, preparation time range [1–5] min, travel time range [5–10] min, clearance time [15–30] min, and total time [15–45] min. These time ranges all contained most of the data for each time. These results indicate that although a number of extreme situations occurred, we could predict 86% preparation time, 56% travel time, 23.58% clearance time, and 55.79% total time with a MAPE value of less than 0.5. However, RMSE and MAPE indicate unreasonable prediction for longer or shorter ranges than the average range, indicating that, similar to previous studies [12], the developed models cannot effectively predict the extreme value. Particularly for extremely short ranges, the MAPE is largest for clearance time and total time that are within [1–15] min.

Table 7 shows the MAE, RMSE, and MAPE calculation results of total time for predicting most incidents in which the extreme values were removed.

As shown in Table 7, we can reasonably predict total time and the shortest time phase.

Another measure of prediction effectiveness is attributed to a certain tolerance of the prediction error. Knowing the percentage of predictions that are within a certain tolerance of their actual duration times is important. Three tolerance values, namely, 15, 30, and 60 min, were used to analyze the prediction result for clearance time and total time.  Table 8 shows the certain tolerance of the prediction error of clearance time and total time.

As shown in Table 8, we can predict 95% of the data with an absolute error of less than 60 min for clearance time and total time. Up to 73% of the data for clearance time had an error of less than 15 min, and 71% of the data for total time had an error of less than 15 min. We can thus predict these times with reasonable accuracy. A number of extreme values have occurred which we cannot predict accurately. For example, the longest total time in the data was 341 min, and we predicted it as 35.8 min. The longest and shortest times in the date reduced the MAPE in our study. Tables 5 and 6 show that a number of outliers with a larger prediction error existed, which may be the result of the following: (1) the traffic incident duration time was significantly different based on the individual differences of traffic incident response teams in clearing similar incidents, as well as the different attitudes of the drivers to similar incidents; (2) the data used in this study were mainly based on the information from the traffic incident report and dispatch system. This information is usually brief and does not include detailed information that can be obtained during the incident treatment and can affect the traffic incident duration time.

## 6. Conclusions and Recommendations

This study proposed different hazard-based models, including a general model and a flexible model, to investigate the factors that affect each incident duration phase in the third ring road of Beijing. The model estimation results show that various factors significantly affect different incident duration phases, including shift of day, season, incident character, incident type, distance from city center, and congestion level. Moreover, these findings present incident management operators with recommendations for reducing different incident duration phases.

This study found that the best distributions for different incident duration phases varied, which is in accordance with the findings in previous studies. Although the flexible model had flexibility, it was not the optimal for all cases. Although the flexible model was a semiparametric model and its incident duration time was fit for some distributions, this model did not perform as well as the parametric distribution model.

The prediction result shows that, for most incidents, we can obtain a reasonable prediction result. However, in extreme incidents, the prediction error is unacceptable. The large perdition errors for some outliers may be due to the following issues: (1) the individual differences among traffic incident response teams or the drivers involved in similar traffic incidents; (2) the limited information about the incident because the developed models were implemented at the moment of incident notification and were based on the initial information reported to the traffic control center.

Overall, the proposed models can be used in traffic incident management to predict traffic incident duration based on the initial information of incident reported to the traffic control center. These predictions would be helpful for timely traffic management decision making and real-time traffic operation. Future works should consider including more variables for different traffic incident management phases. Moreover, further study is necessary to apply the results of this study into a prediction system that can help traffic operators make decisions.

## Figures and Tables

**Table 1:** Statistics information of the incident dataset.

Duration phase	Number of incidents	Minimum	Maximum	Mean	Std. deviation	Variance	Skewness	Kurtosis
Preparation time	2851	1	40	3.48	2.39	5.73	5.36	55.91
Travel time	2851	1	245	6.33	7.43	55.22	19.86	589.69
Clearance time	2851	1	339	23.40	33.46	1119.68	4.10	22.82
Total time	2851	3	371	33.22	34.83	1213.02	4.05	22.50

**Table 2:** Candidate variables.

Variable	Value
Temporal characteristics	
Peak hour	Binary variable: 1: peak hours (7:00–9:00 and 17:00–19:00); 0: nonpeak hours
Day first shift	Binary variable: 1: 22:00–6:00; 0: 6:00–22:00
Weekday	Binary variable: 1: weekday; 0: weekend
Season	Categorical variable: 1: spring (reference in estimation), 2: summer, 3: autumn, and 4: winter
Incident characteristics	
Incident type	Categorical variable: 1: more common crash (reference in estimation), 2: rear-end crash, 3: crash involving pedestrian or bicycle, 4: collision with stationary object, 5: overturned vehicle, and 6: others
Treatment type	Binary variable: 1: resolved by police, 0: resolved by drivers involved in incident
Number of vehicles involved	Binary variable: 1: 1 or 2, 0: greater than 2
Taxi	Binary variable: 1: incident involving taxi; 0: no taxi
Bus	Binary variable: 1: incident involving bus; 0: no bus
Truck	Categorical variable: 1: incident involving small truck, 2: incident involving large truck, 0: no truck (reference in estimation)
Geographic characteristics	
Distance	Continuous variable: distance from city center, unit: km
Others	
Congestion	Binary variable: 1: congested traffic condition, 0: noncongested traffic condition
Preparation time	Continuous variable for travel time and clearance time analysis
Travel time	Continuous variable for clearance time analysis

**Table 3:** Different BIC values for each model.

	Preparation time	Travel time	Clearance time	Total time
Weibull	6607.8	6905.507	9341.794	7356.691
Log-normal	4047.376	5458.285	**8971.931**	6066.565
Log-logistic	3978.691	5427.53	9061.068	6043.001
Generalized gamma	**3775.382**	—	8979.707	**5910.645**
Weibull (frailty)	—	—	9170.154	7364.646
Log-normal (frailty)	3804.348	5466.241	8979.886	5923.54
Log-logistic (frailty)	3917.083	5435.485	9069.023	6005.922
Flexible parametric (df1)	5346.534	5922.993	9309.613	6996.767
Flexible parametric (df2)	—	5395.482	9083.092	—
Flexible parametric (df3)	3860.225	5398.458	9064.663	—
Flexible parametric (df4)	3844.671	—	8987.45	5963.48
Flexible parametric (df5)	3838.949	**5392.066**	8979.638	5967.286
Flexible parametric (df6)	3838.504	5396.527	8977.687	5973.956
Flexible parametric (df7)	3844.429	5399.001	8974.994	5980.894
Flexible parametric (df8)	3850.159	5400.531	8974.215	5987.964
Flexible parametric (df9)	3858.68	5394.331	8974.011	5993.911
Flexible parametric (df10)	3865.634	5399.004	8974.629	5999.218

—: the distribution was not fit for the dataset.

**Table 4:** Regression coefficients of different factors and the percent change for each incident.

Variable	Preparation time		Travel time		Clearance time		Total time	
	Parameter estimation	Percent change (%)	Parameter estimation	Percent change (%)	Parameter estimation	Percent change (%)	Parameter estimation	Percent change (%)
Best model	Generalized gamma		Flexible parametric (df5)		Log-normal		Generalized gamma	
Peak hour	—	—	—	—	—	—	—	—
Day first shift	—	—	−0.691 (−7.61)	−49.89	0.553 (5.41)	73.84	0.396 (6.96)	48.58
Weekday	—	—	—	—	—	—	—	—
Summer (reference: spring)	0.125 (5.35)^*^	13.31	—	—	—	—	—	—
Autumn (reference: spring)	0.156 (6.15)	16.88	−0.171 (−2.81)		—	—	—	—
Winter (reference: spring)	—	—	—	—	—	—	−0.075 (−2.25)	−7.22
Rear-end (reference: more common collision)	—	—	—	—	—	—	—	—
Bike (people) included (reference: more common collision)	—	—	−0.626 (−2.97)	−46.52	—	—	0.455 (3.46)	57.61
Collision with stationary object (reference: more common collision)	—	—	−0.302 (−2.08)	−26.06	—	—	0.202 (2.20)	22.38
Overturned vehicle (reference: more common collision)	−0.334 (−2.38)	−28.39	—	—	0.967 (2.54)	163.00	—	—
Fire (reference: more common collision)	—	—	—	—	—	—	—	—
Vehicle number	—	—	—	—	—	—	—	—
Taxi	0.045 (2.20)	4.60	0.109 (2.20)	11.51	—	—	—	—
Bus	—	—	0.125 (2.01)	13.31	—	—	—	—
Truck	—	—	—	—	—	—	—	—
Distance	−0.035 (−4.23)	−3.43	—	—	0.255 (10.71)	29.04	0.135 (10.03)	14.45
Congestion	−0.060 (−3.64)	−5.82	—	—	0.174 (3.92)	19.00	0.106 (4.36)	11.18
Preparation time	NA	NA	—	—	−0.017 (−2.05)	−1.68	NA	NA
Travel time	NA	NA	NA	NA	—	—	NA	NA

NA: The factor was not used in the model.

—: The factor was not significant at 95% level of significance.

∗: If the table cell included numbers, which means the variables are statistically significant at a 95% confidence level.

(*·*): The number in () was the statistical magnitude for each parameter estimation.

Other explanation: The numbers in the parameter estimation column indicate the regression coefficients of different factors.

The numbers in the percent change column indicate the factor effect on each incident phase. For the AFT model, the number indicates the percent change in time. For the flexible parametric model, the number indicates the percent change in hazard rate.

**Table 5:** MAE, RMSE, and MAPE for prediction of preparation time and travel time.

Time range	Preparation time					Travel time				
	MAE	RMSE	MAPE	*N*	Percent	MAE	RMSE	MAPE	*N*	Percent
1–5	0.77	0.93	0.32	819	86.21%	2.73	3.09	1.21	405	42.63%
5–10	3.54	3.82	0.53	111	11.68%	1.37	1.82	0.18	427	44.95%
10–20	11.20	11.58	0.78	17	1.79%	6.29	6.91	0.47	105	11.05%
>20	27.27	29.20	0.89	3	0.32%	16.09	16.74	0.67	13	1.37%

Total	1.37	2.75	0.35	950	100.00%	2.69	3.83	0.66	950	100.00%

**Table 6:** MAE, RMSE, and MAPE for prediction of clearance time and total time.

Time range	Clearance time					Total time				
	MAE	RMSE	MAPE	*N*	Percent	MAE	RMSE	MAPE	*N*	Percent
1–15	6.22	7.86	2.16	512	53.89%	10.73	11.91	1.08	250	26.32%
15–30	8.99	10.54	0.40	224	23.58%	7.04	5.79	0.21	358	37.68%
30–45	22.41	23.32	0.61	103	10.84%	8.43	14.42	0.36	172	18.11%
45–60	36.48	37.44	0.70	37	3.89%	27.06	28.08	0.52	71	7.47%
60–120	65.53	67.91	0.80	50	5.26%	53.59	55.33	0.67	69	7.26%
>120	171.85	173.53	0.91	24	2.53%	158.27	168.24	0.85	30	3.16%

Total	17.11	35.27	1.42	950	100.00%	17.84	35.51	0.54	950	100.00%

**Table 7:** MAE, RMSE, and MAPE for prediction of total time of most incidents.

Time range	Total time				
	MAE	RMSE	MAPE	*N*	Percent
>15	20.27	40.76	0.35	700	73.68%

**Table 8:** Certain tolerance of the prediction error.

Certain tolerance	Clearance time		Total time	
	Value	Percent	Value	Percent
15 minutes	691	0.73	678	0.71
30 minutes	835	0.88	825	0.87
60 minutes	901	0.95	898	0.95
